# Dietary Hemp (*Cannabis sativa* L.) Products Enhance Egg Yolk Omega-3 Fatty Acids and Color Without Compromising Laying-Hen Performance: A Meta-Analysis

**DOI:** 10.3390/ani15142062

**Published:** 2025-07-12

**Authors:** Yusup Sopian, Panneepa Sivapirunthep, Anuraga Jayanegara, Chanporn Chaosap

**Affiliations:** 1 Doctoral Program in Innovative Tropical Agriculture, School of Industrial Education and Technology, King Mongkut’s Institute of Technology Ladkrabang, Bangkok 10520, Thailand; 65036096@kmitl.ac.th; 2 Department of Agricultural Education, School of Industrial Education and Technology, King Mongkut’s Institute of Technology Ladkrabang, Bangkok 10520, Thailand; panneepa.si@kmitl.ac.th; 3 Department of Animal Nutrition and Feed Technology, Faculty of Animal Science, IPB University, Bogor 16680, Indonesia; anuragaja@apps.ipb.ac.id

**Keywords:** production performance, hen-day production, egg weight, egg quality

## Abstract

Hemp (*Cannabis sativa* L.) products have gained attention in poultry nutrition for their rich content of polyunsaturated fatty acids (PUFAs), bioactive compounds, and potential functional benefits. However, findings on their impact on laying-hen performance, egg quality, and yolk fatty acid profiles have been inconsistent. This meta-analysis aimed to evaluate the effects of dietary hemp products on laying-hen performance, egg quality traits, and yolk fatty acid composition, while exploring potential sources of heterogeneity across studies. A comprehensive literature search identified 21 studies that met the inclusion criteria. A random-effects model was used to calculate standardized mean differences (SMDs) with 95% confidence intervals (CIs) for various outcomes, including production performance, egg quality, and yolk fatty acid profiles. Subgroup and meta-regression analyses assessed the influence of factors such as inclusion level, hen age, and hemp product type. The results showed that hemp supplementation had no significant effect on hen-day production, egg mass, feed conversion ratio, or feed intake. However, yolk redness (SMD = 4.40; 95% CI: 2.46, 6.33; *p* < 0.001) and yellowness (SMD = 4.45; 95% CI: 2.75, 6.16; *p* < 0.001) were significantly enhanced. Hemp feeding also increased n-3 PUFA levels in egg yolk, including C18:3n3, C20:5n3, and C22:6n3, while reducing saturated and monounsaturated fatty acids. Subgroup analysis indicated that inclusion levels > 10% and hen age ≤ 25 weeks were associated with slight reductions in hen-day production, whereas older hens (>25 weeks) showed increased egg weight. In conclusion, hemp products can enrich yolk pigmentation and n-3 PUFA content in eggs, supporting the production of functional, value-added eggs without compromising the laying-hen performance.

## Simple Summary

Hemp (*Cannabis sativa* L.), a plant known for its healthy fats and natural compounds, is being explored as an alternative feed ingredient, especially for egg-laying hens. This study reviewed results from 21 earlier experiments to understand how feeding hemp to hens affects their performance, the quality of their eggs, and the types of fats found in the yolk. The results found that adding hemp products to the diet did not affect hen performance. However, it did make the egg yolks a richer yellow and red color, and it increased the amount of healthy omega-3 fats, which are good for human health. These findings suggest that hemp can help produce eggs with added health benefits and a more appealing yolk color. However, farmers and feed producers need to be careful with how much hemp they use, especially for younger hens. More research is needed to find the best ways to use hemp in poultry diets to benefit both animals and consumers.

## 1. Introduction

Hemp (*Cannabis sativa* L.) has gained increasing attention as a feed ingredient in animal nutrition, driven by its unique nutritional composition and potential to enhance the quality of animal-derived products. Hemp products, including hempseed (HS), hempseed cake (HC), hempseed oil (HO), and other co-products (HCP), are rich in polyunsaturated fatty acids (PUFAs), high-quality protein, fiber, minerals, and bioactive compounds such as cannabinoids, tocopherols, and phenolics [1,2]. These components hold promises for enhancing the nutritional value of animal products, including eggs, while also supporting animal health and welfare [1]. Although hemp cultivation was historically restricted due to regulatory concerns about its psychoactive constituents, particularly tetrahydrocannabinol (THC), recent policy reforms have allowed for the reintroduction of industrial hemp, which is defined by a low THC content (<0.3%), into agricultural systems [3]. This renewed availability has sparked growing interest in the use of hemp-derived ingredients in livestock and poultry diets.

In laying-hen production, nutritional interventions that improve egg quality and nutrient composition are of increasing interest to both producers and consumers. Hemp products, particularly rich in α-linolenic acid (ALA, C18:3n3) and other essential fatty acids, offer a promising approach for enriching egg yolks with beneficial long-chain n-3 PUFAs, such as eicosapentaenoic acid (EPA, C20:5n3) and docosahexaenoic acid (DHA, C22:6n3) [1]. These fatty acids are well-known for their positive effects on human health, including cardiovascular protection and anti-inflammatory properties [4]. Additionally, the incorporation of hemp products into poultry diets may influence yolk pigmentation, an attribute valued by both producers and consumers.

Despite these promising attributes, research findings on the effects of hemp supplementation in laying hens remain inconsistent. While several studies report improved yolk fatty acid profiles and enhanced yolk pigmentation [5,6,7], others raise concerns about potential reductions in key performance metrics, such as egg production and feed efficiency, particularly at higher inclusion levels [8,9]. A critical limitation of the existing body of research is the variability in experimental design, small sample sizes, short study durations, and the inconsistent reporting of outcomes, which collectively hinder the ability to draw generalizable conclusions. Moreover, the other confounding factors such as breed, age, and environmental conditions can significantly influence the observed effects [10,11,12]. These methodological weaknesses necessitate a rigorous and systematic evaluation of the current evidence.

To address these gaps, a meta-analysis was conducted to synthesize the effects of hemp product supplementation on laying-hen performance, egg quality, and yolk fatty acid composition. The analysis included only peer-reviewed, controlled experimental studies with quantifiable outcomes. Eligible studies were required to report at least one performance or egg quality parameter, along with corresponding sample sizes and measures of variance.

Although the available body of literature is still relatively limited, a meta-analytic approach remains valuable for quantifying overall effect sizes, exploring sources of heterogeneity through subgroup and meta-regression analyses, and identifying consistent patterns or areas of uncertainty [13]. Importantly, this synthesis also provides a foundation for future research and practical dietary recommendations. Limitations related to study quantity and quality are acknowledged and discussed to ensure transparent interpretation of the findings.

## 2. Materials and Methods

### 2.1. Literature Search and Study Selection

**Figure 1:**
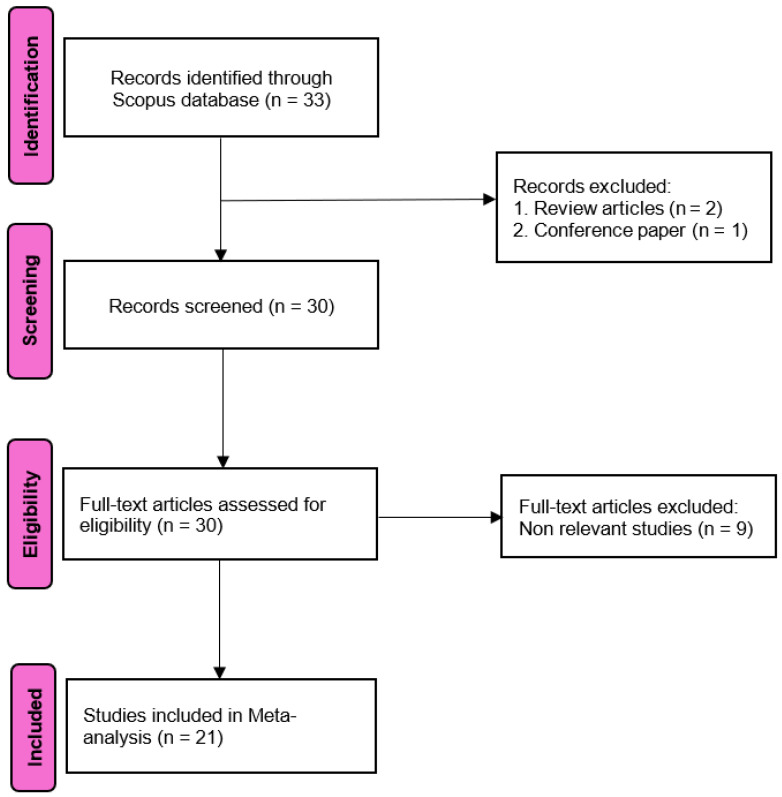
The selection process of the studies using the PRISMA method.

A comprehensive list of studies examining the use of hemp products in laying-hen diets was compiled in January 2025 from the Scopus database using search terms such as “hemp,” “seed,” “oil,” “cake,” and “hen,” combined with Boolean operators (AND/OR) without limiting publication years. Additionally, the reference lists of relevant articles were manually screened to identify further eligible studies. Studies involving other animal species or review articles were excluded. The study selection process is illustrated in Figure 1. The entirety of the database development process was in accordance with the Preferred Reporting Items for Systematic Reviews and Meta-Analysis (PRISMA) protocol [14]. The PRISMA checklist was used to ensure the inclusion of all relevant information in the analysis (Supplementary Materials Data S1).

To be included in the meta-analysis, studies had to meet the following criteria:

(i) The conducted experiments evaluated the effects of hemp products on laying hens.
(ii) Be published in English.
(iii) Report at least one relevant outcome measure, including hen-day production, egg mass, feed conversion ratio (FCR), feed intake (FI), egg weight (EW), albumen weight (AW), yolk cholesterol (YC), yolk weight (YW), shell weight (SW), Haugh unit (HU), eggshell thickness (EST), yolk color (lightness (L*), redness (a*), and yellowness (b*)), or yolk fatty acid profiles.
(iv) Provide mean values, sample sizes (number of birds per treatment group), and a measure of variability, either in numerical or graphical form.

### 2.2. Data Extraction

The following information was extracted from each study: publication year, country, hemp product type, inclusion levels (%), hen strain, hen age (weeks), feeding duration (weeks), sample sizes, means, and standard deviations (or standard errors) for the outcomes of interest.

### 2.3. Data Analysis

The meta-analysis was performed using a random-effects model with the Open Meta-analyst for Ecology and Evolution (OpenMEE) software [15], applying the DerSimonian–Laird method [16]. Effect sizes were expressed as standardized mean differences (SMDs) with 95% confidence intervals (CIs), following the guidelines of Koricheva et al. [17]. A significance level of 5% was used for all pooled estimates.

The effect size calculation was based on the standardized mean difference of Hedges’ d using the following formula [18].

$$d=\frac{\left(X^{e}-X^{c}\right)}{S}\;J$$

where X^e^ is the means of the hemp products group; X^c^ is the control group; S the pooled standard deviation; and J the correction factor for the small sample size, i.e.,:

$$J=1-\frac{3}{{(4(N}^{e}+N^{c}))-1}$$

$$S=\sqrt{\frac{{(N}^{e}-1)({S^{e})}^{2}+\left(N^{c}-1\right)({S^{c})}^{2}}{N^{e}+N^{c}-2}}$$

where N^e^ is the sample size of the hemp products group; N^c^ is the sample size of the control group; S^e^ is the standard deviation of the hemp products group; and S^c^ is the standard deviation of the control group.

The mathematical modeling of the one-way random effect is as follows:

y_i_ = θ + v_i_+ ε_i_

where y_i_ is the value of the effect size (in Hedge’s d); θ the i-th observation (the general parameter of the combined effect size; v_i_ the real variation in the effect size; and εi the error of the i-th observation.

The estimation of the variance between studies (τ^2^) was based on the DerSimonian and Laird [16] method:

$$\tau^{2}=\frac{Q-df}{C}$$

where Q is Cochran’s Q statistic (the weighted sum of squared differences), df is the degrees of freedom, and C is a scaling factor that reflects the variability and distribution of the inverse-variance weights assigned to each study.

### 2.4. Heterogeneity Assessment

Heterogeneity among studies was assessed using the I^2^ statistic [19]. The I^2^ was classified into four categories of heterogeneity according to Borenstein et al. [20] as follows: no evidence of heterogeneity (0 < I^2^ ≤ 25%), low (25% < I^2^ ≤ 50%), moderate (50% < I^2^ ≤ 75%), and high (I^2^ ≥ 75%).

Formula for I^2^:

$$I^{2}=\frac{Q-df}{C}\;\times \;100$$

where Q is Cochran’s Q statistic and df is the degrees of freedom.

### 2.5. Subgroup and Meta-Regression Analyses

To explore potential sources of heterogeneity, subgroup analyses were conducted on five main categories: (1) hemp products (hemp seed, hemp oil, and hemp cake)—the other co-product was excluded due to the low number of studies including it; (2) inclusion levels (≤10% and >10%); (3) hen age (≤25 and >25 wks); (4) feeding duration (≤10 and >10 wks); and (5) layer strain (Bovan and Lohmann). However, subgroup analyses were only conducted in the largest sample size each category. Therefore, the subgroup was evaluated in hen-day production (performance), egg weight (egg quality), and C22:6n3 fatty acid content (yolk fatty acid) [21]. Subgroups with fewer than three studies were excluded from the analysis due to insufficient statistical power [22]. A meta-regression analysis was also conducted to examine the relationship between hemp product inclusion and selected outcomes [23].

### 2.6. Publication Bias Assessment

Publication bias was evaluated using Rosenberg’s fail-safe number (Nfs), which estimates the number of hypothetical null-effect studies required to render the meta-analysis non-significant. According to Rosenthal’s threshold, Nfs > 5n + 10 was considered robust. In addition, funnel plots were visually inspected for asymmetry [17,24].

Formula for Nfs:

$$Nfs=\frac{\sum Z^{2}-(k){\left(Z_{critical}\right)}^{2}}{{\left(Z_{critical}\right)}^{2}}$$

where ∑Z = the sum of Z-values from the included studies (converted from effect sizes and variances), Z_critical_ = 1.645 for one-tailed test at α = 0.05 (or 1.96 for two-tailed), and k = number of studies in the meta-analysis.

## 3. Results

### 3.1. Study Characteristics

Table 1 summarizes the characteristics of the 21 primary research articles that met the inclusion criteria. These studies were published between 2005 and 2025 and were conducted across nine countries on four continents. Seven different strains of laying hens, aged 19 to 52 weeks, were fed diets containing 0–30% hemp products for durations ranging from 4 to 24 weeks.

### 3.2. Laying-Hen Performance

**Figure 2:**
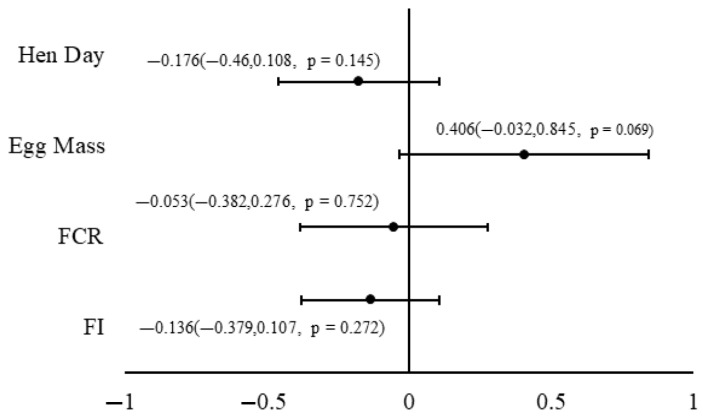
A forest plot of the effects of hemp product supplementation on laying-hen performance outcomes (SMD, standardized mean differences), including feed conversion ratio (FCR), feed intake (FI), hen-day egg production, and egg weight [6,7,8,9,25,26,27,28,29,32,35,36,38,40].

As shown in Figure 2, the inclusion of hemp products in the diet had no significant effect (*p* > 0.05) on production performance parameters, including hen-day egg production, egg mass, FCR, and FI.

### 3.3. Egg Quality

Table 2 shows that egg quality traits such as egg weight (EW), albumen weight (AW), yolk cholesterol (YC), yolk weight (YW), shell weight (SW), Haugh unit (HU), eggshell thickness (EST), and yolk lightness (L*) did not differ significantly between hens fed hemp-containing diets and those on control diets. However, yolk redness (SMD = 4.396; 95% CI: 2.459, 6.334; *p* < 0.001) and yellowness (SMD = 4.452; 95% CI: 2.745, 6.158; *p* < 0.001) were significantly higher in hens fed diets containing hemp products.

**Table 2:** Effects of hemp products on egg quality [5,6,7,8,9,25,26,27,28,29,32,34,36,38,39,40].

Variable	NC	Estimate (SMD)	Lower Bound	Upper Bound	Std. Error	*p*-Value	τ^2^	Q	Het. *p*-Value	I^2^
EW	33	0.24	−0.13	0.61	0.189	0.203	0.882	178.058	<0.001	82.028
AW	17	0.139	−0.181	0.459	0.163	0.395	0.203	31.159	0.013	48.65
YW	19	0.126	−0.213	0.465	0.173	0.466	0.296	41.348	0.001	56.467
YC	9	−0.947	−2.475	0.581	0.779	0.224	3.375	73.985	<0.001	89.187
HU	11	0.36	−0.093	0.814	0.231	0.12	0.303	21.041	0.021	52.474
SW	16	0.008	−0.454	0.47	0.236	0.972	0.641	67.378	<0.001	77.738
EST	21	−0.376	−0.934	0.182	0.285	0.187	1.409	160.697	<0.001	87.554
L*	9	−1.449	−3.644	0.746	1.12	0.196	10.627	195.928	<0.001	95.917
a*	15	4.396	2.459	6.334	0.989	<0.001	11.405	345.784	<0.001	95.951
b*	15	4.452	2.745	6.158	0.871	<0.001	8.793	300.803	<0.001	95.346

NC: number of sample size comparisons; SMD: standardized mean differences; Std. error: standard error; τ^2^: estimate of variance between studies in a random effects meta-analysis; Q: study homogeneity; Het. *p*-Value: heterogeneity *p*-value; I^2^: percentage of variation across studies due to heterogeneity; EW: egg weight; AW: albumen weight; YC: yolk cholesterol; YW: yolk weight; SW: shell weight; HU: Haugh unit; EST: egg shell thickness; L*: lightness; a*: redness; and b*: yellowness.

### 3.4. Egg Yolk Fatty Acid Profiles

Table 3 presents the effects of dietary hemp products on egg yolk fatty acid composition. Hens fed hemp-containing diets exhibited significantly lower levels of C14:0, C16:0, C16:1, C18:1, C20:4n6, and total MUFA (*p* < 0.001), compared to control hens. In contrast, levels of C18:0, C18:2, C18:3n3, C18:3n6, C20:5n3, C22:5n3, C22:6n3, total n-3, total n-6, and total PUFA were significantly higher (*p* < 0.01) in the hemp-fed groups. No significant differences were found for C12:0 or total SFA content (*p* > 0.05).

**Table 3:** Effects of hemp products on egg yolk fatty acid profiles [5,6,7,8,25,26,27,28,30,31,32,33,34,35,37,38,39,40].

Variable	NC	Estimate (SMD)	Lower Bound	Upper Bound	Std. Error	*p*-Value	τ^2^	Q	Het. *p*-Value	I^2^
C12:0	13	1.112	−0.371	2.596	0.757	0.142	5.05	115.407	<0.001	91.335
C14:0	37	−0.905	−1.36	−0.45	0.232	<0.001	1.509	190.081	<0.001	81.061
C16:0	48	−1.588	−2.08	−1.097	0.251	<0.001	2.287	356.074	<0.001	86.801
C16:1	46	−1.373	−1.881	−0.865	0.259	<0.001	2.399	363.798	<0.001	87.631
C18:0	48	0.607	0.118	1.096	0.25	0.015	2.338	368.46	<0.001	87.244
C18:1	48	−1.905	−2.373	−1.437	0.239	<0.001	2.045	311.562	<0.001	84.915
C18:2	48	1.305	0.846	1.765	0.235	<0.001	1.886	328.457	<0.001	85.691
C18:3n3	48	5.595	4.724	6.466	0.444	<0.001	6.891	448.988	<0.001	89.532
C18:3n6	37	2.178	1.594	2.763	0.298	<0.001	2.488	259.954	<0.001	86.151
C20:4n6	33	−1.071	−1.558	−0.584	0.248	<0.001	1.62	192.58	<0.001	83.384
C20:5n3	38	4.573	3.829	5.317	0.38	<0.001	3.851	262.648	<0.001	86.293
C22:5n3	25	2.553	1.918	3.189	0.324	<0.001	2.102	150.699	<0.001	84.074
C22:6n3	41	3.894	3.23	4.557	0.339	<0.001	3.674	302.581	<0.001	86.78
∑ n-3	35	5.132	4.294	5.97	0.428	<0.001	4.779	245.575	<0.001	86.155
∑ n-6	30	1.068	0.386	1.75	0.348	0.002	2.603	243.662	<0.001	88.098
∑ SFA	28	−0.363	−0.764	0.037	0.204	0.076	0.851	112.27	<0.001	75.951
∑ MUFA	31	−2.694	−3.425	−1.962	0.373	<0.001	3.051	224.53	<0.001	86.639
∑ PUFA	25	3.121	1.796	4.445	0.676	<0.001	8.083	376.35	<0.001	93.623

NC: number of sample size comparisons, SMD: standardized mean differences, Std. error: standard error; τ^2^: estimate of variance between studies in a random effects meta-analysis, Q: study homogeneity, Het. *p*-Value: heterogeneity *p*-value; I^2^: percentage of variation across studies due to heterogeneity; n-3: ω-3 fatty acids, n-6: ω-6 fatty acids, SFA; saturated fatty acid, MUFA: monosaturated fatty acid, PUFA: polyunsaturated fatty acid.

### 3.5. Subgroup Analysis

The results of the subgroup analysis (Table 4) revealed that inclusion levels above 10% (SMD = −0.508; 95% CI: −0.996, −0.019; *p* < 0.05) and hen age ≤ 25 weeks (SMD = −0.279; 95% CI: −0.547, −0.011; *p* < 0.05) were associated with reduced hen-day production. Conversely, hens older than 25 weeks showed a significant increase in egg weight (SMD = 1.647; 95% CI: 0.369, 2.925; *p* < 0.05). All moderator variables in the subgroup analysis were significantly associated with increased levels of C22:6n3 fatty acid (*p* < 0.001).

**Table 4:** A subgroup analysis of the effect of hemp products on hen day, egg weight, and docosahexaenoic acid (DHA) [5,6,7,8,9,25,26,27,28,29,30,31,32,33,34,35,36,37,38,39,40].

Variable	Moderators	Subgroup	NC	Estimate (SMD)	Lower Bound	Upper Bound	Std. Error	*p*-Value
Hen day	Hemp products	HS	14	−0.135	−0.623	0.353	0.249	0.588
		HC	11	−0.352	−0.78	0.076	0.218	0.107
		HO	9	0.249	−0.31	0.808	0.285	0.383
	Inclusion levels (%)	≤10	25	−0.006	−0.362	0.35	0.181	0.974
		>10	12	−0.508	−0.996	−0.019	0.249	0.042
	Hen age (wks)	≤25	23	−0.279	−0.547	−0.011	0.137	0.042
		>25	14	0.153	−0.522	0.829	0.345	0.656
	Feeding duration (wks)	≤10	16	−0.352	−0.909	0.205	0.284	0.216
		>10	21	−0.072	−0.393	0.249	0.164	0.66
	Layer strain	Bovan	8	−0.487	−1.002	0.029	0.263	0.064
		Lohmann	20	−0.308	−0.715	0.099	0.208	0.138
Egg weight	Hemp products	HS	14	0.634	−0.139	1.407	0.394	0.108
		HC	8	−0.047	−0.537	0.443	0.25	0.851
		HO	8	0.088	−0.66	0.836	0.382	0.817
	Inclusion levels (%)	≤10	22	0.426	−0.11	0.962	0.274	0.119
		>10	11	0.022	−0.371	0.415	0.2	0.912
	Hen age (wks)	≤25	23	−0.08	−0.308	0.149	0.117	0.495
		>25	10	1.647	0.369	2.925	0.652	0.012
	Feeding duration (wks)	≤10	12	0.258	−0.339	0.854	0.304	0.397
		>10	21	0.238	−0.237	0.713	0.242	0.326
	Layer strain	Bovan	8	0.171	−0.202	0.544	0.19	0.368
		Lohmann	20	−0.08	−0.545	0.385	0.237	0.736
C22:6n3 DHA	Hemp products	HS	20	4.167	2.963	5.371	0.614	<0.001
		HC	5	2.75	1.89	3.611	0.439	<0.001
		HO	13	4.237	3.122	5.353	0.569	<0.001
	Inclusion levels (%)	≤10	23	3.663	2.947	4.379	0.365	<0.001
		>10	18	4.475	3.151	5.798	0.675	<0.001
	Hen age (wks)	≤25	31	3.794	3.153	4.436	0.327	<0.001
		>25	10	4.707	2.844	6.569	0.95	<0.001
	Feeding duration (wks)	≤10	16	3.076	2.141	4.01	0.477	<0.001
		>10	25	4.329	3.425	5.233	0.461	<0.001
	Layer strain	Bovan	13	4.347	3.878	4.815	0.239	<0.001
		Lohmann	19	3.977	2.966	4.988	0.516	<0.001

NC: number of sample size comparisons; SMD: standardized mean differences; HS: hempseed; HC: hempseed cake; and HO: hempseed oil.

### 3.6. Meta-Regression and Publication Bias

**Figure 3:**
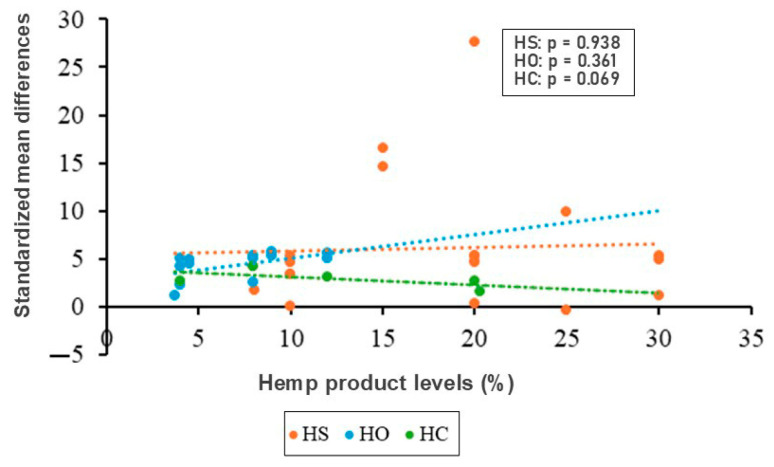
Meta-regression between C22:6n3 (DHA) and hemp product levels. HS (hempseed), HC (hempseed cake), and HO (hempseed oil) [5,6,7,8,25,26,27,28,30,31,33,34,37,38,39,40].

**Figure 4:**
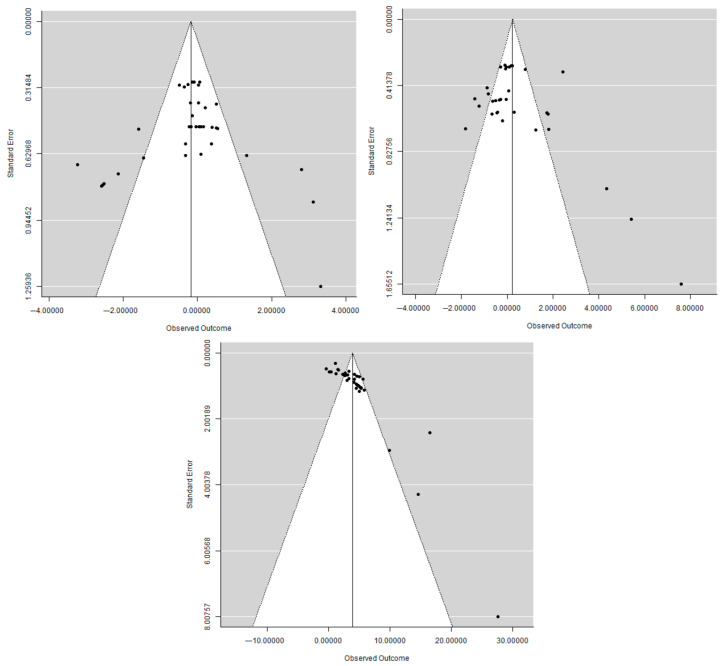
Funnel plot effect of hemp product supplementation on hen day, egg weight, and C22:6n3 (DHA).

Meta-regression analysis showed no significant association between the level of dietary hemp product inclusion and C22:6n3 content (Figure 3). Evidence of publication bias was observed for hen-day production, egg weight, and C22:6n3, as indicated by Rosenberg’s fail-safe number (Table 5). Funnel plots for these outcomes (Figure 4) appeared nearly symmetrical, suggesting minimal bias in the included studies.

## 4. Discussion

This meta-analysis provides a comprehensive synthesis of 21 experimental studies investigating the effects of dietary hemp products on laying-hen performance, egg quality, and yolk fatty acid profiles. Overall, our findings confirm the nutritional potential of hemp products, particularly in enriching yolk pigmentation and omega-3 fatty acids, while also highlighting conditions under which performance outcomes may be negatively affected.

Heterogeneity among the included studies was assessed using Cochran’s Q test and quantified using the I^2^ statistic. Several outcome measures demonstrated moderate to high heterogeneity, indicating substantial variability across studies. To investigate potential sources of this heterogeneity, subgroup analyses were performed based on hemp product type, inclusion level, hen age, feeding duration, and layer strain. These subgroup analyses helped to partially explain the observed variation. Additionally, meta-regression analysis was conducted to further identify factors contributing to the heterogeneity across studies.

### 4.1. Impact on Laying-Hen Performance and Egg Quality

The pooled results indicate that hemp supplementation had no significant effect on hen-day egg production, egg mass, feed intake, or feed conversion ratio, consistent with individual reports by Neijat et al. [29] and Mierlita et al. [28], who observed similar neutrality in performance outcomes at moderate inclusion levels. However, subgroup analysis revealed that inclusion levels > 10% and use in hens ≤ 25 weeks of age were associated with reduced egg production, aligning with findings from Öztürk et al. [8] and Taaifi et al. [9], who noted suppressed productivity when diets included high levels of hemp cake or seed. These results suggest that younger hens may be more sensitive to dietary changes and that high inclusion levels could introduce anti-nutritional effects or disrupt nutrient balance.

In contrast, older hens (>25 weeks) exhibited a significant increase in egg weight, particularly with moderate hemp inclusion. This finding supports reports by Mierlita et al. [28] and Konca et al. [26], who found improvements in egg weight and yolk mass among mature laying hens supplemented with hempseed or oil.

Regarding egg quality, most internal and shell traits remained unaffected, though a consistent enhancement in yolk redness (a*) and yellowness (b*) was observed across studies. This effect was especially pronounced with hemp oil and hempseed, likely due to their natural pigment content such as carotenoids and chlorophylls [5,7,39]. This pigmentation effect was weaker with hempseed cake, which undergoes oil extraction, likely reducing pigment availability. These results suggest a product-specific effect on yolk coloration, which has practical relevance for consumer preference and egg grading.

### 4.2. Enrichment of Yolk Fatty Acid Profile

One of the most consistent outcomes was the significant enhancement of egg yolk n-3 polyunsaturated fatty acids, including ALA (C18:3n3), EPA (C20:5n3), and DHA (C22:6n3), following hemp supplementation. This finding was robust across all hemp product types and hen ages, confirming prior observations by Gakhar et al. [25], Goldberg et al. [5], and Neijat et al. [29,31]. Our subgroup analysis showed that all product types, especially hemp oil, were effective in enhancing DHA content, supporting the idea that hemp-derived lipids are efficiently transferred into egg yolk.

Although some individual studies suggested a dose-dependent increase in DHA (e.g., Neijat et al. [30]), our meta-regression did not show a statistically significant linear relationship between inclusion level and C22:6n3 enrichment, suggesting other factors (e.g., fatty acid conversion efficiency, oil processing, or hen metabolism) may mediate this effect. Nonetheless, the consistently large effect sizes, reflected by SMD values of ≥0.8 [41], indicate that even moderate levels of hemp supplementation can substantially enhance the functional value of eggs.

The ability of hemp products to enhance the n-3 PUFA content of egg yolk presents a valuable strategy for producing functional health-promoting eggs aligned with consumer demand for omega-3-enriched foods. Omega-3 fatty acids, including ALA, EPA, and DHA, are essential for human health [42]. They support cardiovascular function by lowering triglycerides, reducing blood pressure, and improving endothelial function [43]. Additionally, omega-3s promote cognitive health [44] and visual function [45], while their anti-inflammatory properties may help manage chronic diseases [46]. Increasing omega-3 intake through diet or supplements is recommended to support overall health and reduce the risk of chronic conditions [47].

### 4.3. Novel Contributions of This Meta-Analysis

This meta-analysis offers several novel contributions to the field of poultry nutrition. It is the first quantitative synthesis to evaluate and compare the effects of different hemp products, including hempseed, hempseed oil, and hempseed cake, on egg quality traits and yolk fatty acid profiles in laying hens. Subgroup analyses revealed distinct product-specific effects, with hemp oil demonstrating greater efficacy in enhancing yolk pigmentation and DHA enrichment compared to hemp cake. The analysis also identified critical thresholds, such as inclusion levels above 10% and supplementation during early laying phases, where productivity may be negatively affected. Collectively, these findings provide practical guidance for optimizing hemp use in layer diets and confirm that hemp-derived feed ingredients can support the production of omega-3 enriched, value-added eggs without compromising overall laying-hen performance when applied under appropriate conditions.

### 4.4. Critical Appraisal and Publication Bias

The robustness of the meta-analysis findings was supported by an assessment of publication bias and a critical appraisal of the included studies. Rosenberg’s fail-safe number (Nfs) was used to estimate the number of missing or unpublished studies with null results required to overturn the observed significant findings. Higher Nfs values indicate greater confidence in the results. In this study, the enrichment of C22:6n3 (DHA) demonstrated very high robustness against potential publication bias, with an Nfs value far exceeding the recommended threshold. In contrast, although the results for hen-day egg production and egg weight were statistically significant, their Nfs values (26 and 56, respectively) were below the suggested robustness thresholds of 85 and 75 (calculated as 5n + 10). These findings should therefore be interpreted with greater caution, as they may be more susceptible to the influence of unpublished negative or null studies. Visual inspection of the funnel plots showed general symmetry, suggesting minimal publication bias overall, although slight asymmetry was noted for hen-day egg production and egg weight, likely due to smaller studies or marginal effects. These observations highlight the importance of continued publication of studies with negative or inconclusive results to ensure a balanced and comprehensive evidence base.

Despite the promising results, several methodological limitations were identified in the included studies. Many trials had small sample sizes, lacked detailed descriptions of randomization procedures and blinding (where applicable), and were of short duration (<10 weeks), limiting the ability to assess long-term effects. Considerable variability in hemp sources, processing methods, and basal diets also contributed to heterogeneity. Moreover, some outcomes had a limited number of comparisons, particularly within subgroup analyses, reducing statistical power.

Future research should prioritize standardized study designs, consistent outcome reporting, and longer feeding durations to enhance the reliability and generalizability of findings on hemp supplementation in laying hens.

## 5. Conclusions

This meta-analysis underscores the potential of hemp-derived products as functional feed ingredients in laying-hen nutrition. Supplementation with hempseed, hempseed cake, or hempseed oil was found to significantly enhance yolk pigmentation and enrich long-chain omega-3 fatty acids, particularly DHA, without adversely affecting key performance metrics such as hen-day egg production, egg mass, or feed conversion ratio when inclusion levels are appropriately managed. Subgroup analyses revealed that inclusion levels above 10% and use in younger hens (≤25 weeks) may negatively impact productivity, whereas older hens exhibited improved egg weight. Among the different hemp product types, hemp oil demonstrated the most pronounced benefits for yolk coloration and fatty acid enrichment. While the analysis is limited by a relatively small number of studies, notable heterogeneity, and occasional gaps in reported data, the findings offer practical insights for the strategic use of hemp products in layer diets and emphasize the need for more standardized, long-duration research to validate and refine these applications.

## Figures and Tables

**Table 1:** The characteristics of the studies included in the meta-analysis.

No	Country	Hemp Products	Inclusion Levels (%)	Hen Strains	HA	FD	Reference
1	Canada	HO, HS	HO: 0, 4, 8, 12; HS: 10, 20	Bovan	19	12	[25]
2	Canada	HO, HS	HO: 0, 4, 8, 12; HS: 10, 20	Bovan	19	12	[5]
3	Canada	HO	0, 4, 8	Lohmann	19	6	[6]
4	Turkey	HO	0, 3.7	Leghorn	42	12	[7]
5	Turkey	HS	0, 15	Lohmann	36	12	[26]
6	Romania	HS, HC	HS: 0, 8.4; HC: 20.32	Tetra-SL	24	10	[27]
7	Romania	HS	0, 8	Tetra-SL	35	8	[28]
8	Canada	HO, HS	HS: 0, 10, 20, 30 HO: 4.5, 9	Lohmann	19	12	[29]
9	Canada	HO, HS	HS: 0, 10, 20, 30; HO: 4.5, 9	Lohmann	19	12	[30]
10	Canada	HO, HS	HS: 0, 10, 20, 30; HO: 4.5, 9	Lohmann	19	12	[31]
11	Turkey	HC	0, 4, 8, 12	Lohmann	50	6	[8]
12	Korea	HO	0, 1.68	Hyline	30	5	[32]
13	Pakistan	HS	0, 25	Hyline	30	5	[33]
14	Pakistan	HS	0, 15, 20, 25	Rhode Island Red	n.a.	4	[34]
15	Canada	HC	0, 5, 10, 20	DeKalb	42	4	[35]
16	Czech	HS	0, 3, 6, 9	Lohmann	52	12	[36]
17	Morocco	HS	0, 10, 20, 30	Lohmann	22	14	[9]
18	Morocco	HS	0, 10, 20, 30	Lohmann	22	14	[37]
19	Germany	HC	5, 10, 15	Lohmann	22	24	[38]
20	Romania	HC	0, 20	Tetra-SL	28	9	[39]
21	Italia	HCP	0, 3, 6, 9	Bovan	21	9	[40]

HS: hempseed, HC: hempseed cake, HO: hempseed oil, and HCP: other hemp co-products were characterized by the presence of leaves, non-standard hempseed, hempseed hulls, and stems; HA: hen age (weeks); FD: feeding duration (weeks).

**Table 5:** Analysis of publication bias.

**Variable**	**Observed Significance**	**Target Significance**	**Nfs**	**No. of** **Study (n)**	**Nfs > [5(n)+ 10]**
Hen day	0.016	0.05	26	15	85
Egg weight	0.004	0.05	56	13	75
C22:6n3	<0.001	0.05	11,547	16	90

Nfs: Rosenberg’s fail-safe number.

## Data Availability

The dataset underlying the reported results is available from the corresponding authors and will be provided upon reasonable request.

## Supplementary Materials

The following supporting information can be downloaded at: https://www.mdpi.com/article/10.3390/ani15142062/s1, Data S1. PRISMA 2020 Checklist 1.

## References

[1] Lanzoni D., Skrivanova E., Pinotti L., Rebucci R., Baldi A., Giromini C. (2024). Review: Nutritional Aspects of Hemp-Based Products and Their Effects on Health and Performance of Monogastric Animals. Animal.

[2] Kamle M., Mahato D.K., Sharma B., Gupta A., Shah A.K., Mahmud M.M.C., Agrawal S., Singh J., Rasane P., Shukla A.C. (2024). Nutraceutical Potential, Phytochemistry of Hemp Seed (*Cannabis sativa* L.) and Its Application in Food and Feed: A Review. Food Chem. Adv..

[3] Mohamed N., House J.D. (2024). Safety and Efficacy of Hemp-Derived Products in Animal Feeds—A Narrative Review. Can J. Anim. Sci..

[4] Kapoor B., Kapoor D., Gautam S., Singh R., Bhardwaj S. (2021). Dietary Polyunsaturated Fatty Acids (PUFAs): Uses and Potential Health Benefits. Curr. Nutr. Rep..

[5] Goldberg E.M., Gakhar N., Ryland D., Aliani M., Gibson R.A., House J.D. (2012). Fatty Acid Profile and Sensory Characteristics of Table Eggs from Laying Hens Fed Hempseed and Hempseed Oil. J. Food Sci..

[6] Jing M., Zhao S., House J.D. (2017). Performance and Tissue Fatty Acid Profile of Broiler Chickens and Laying Hens Fed Hemp Oil and HempOmegaTM. Poult. Sci..

[7] Kanbur G., Göçmen R., Cufadar Y. (2023). A Comparative Study on the Effects of Hemp Seed Oil versus Four Different UFA-Rich Seed Oils’ Dietary Supplementation on Egg Production Performance, Egg Quality, and Yolk Fatty Acids in Laying Hens. Trop. Anim. Health Prod..

[8] Öztürk E., Darmawan A., Özlü Ş., Abacı S.H. (2024). Effects of Dietary Local Hemp Seed Meal as Soybean Meal Alternative on Productive Performance, Egg Quality and Yolk Fatty Acid Composition of Laying Hens. Arch. Anim. Nutr..

[9] Taaifi Y., Belhaj K., Mansouri F., Rbah Y., Elbouanani N., Melhaoui R., Ben Moumen A., Azeroual E., Serghini-Caid H., Elamrani A. (2023). Impact of Cannabis Seed Incorporation in Layer Diet on Productive Performance and Egg Quality Traits. Scientifica.

[10] Kozak A., Kasperek K., Zięba G., Rozempolska-Rucińska I. (2019). Variability of Laying Hen Behaviour Depending on the Breed. Asian-Australas J. Anim. Sci..

[11] Weeks C.A., Lambton S.L., Williams A.G. (2016). Implications for Welfare, Productivity and Sustainability of the Variation in Reported Levels of Mortality for Laying Hen Flocks Kept in Different Housing Systems: A Meta-Analysis of Ten Studies. PLoS ONE.

[12] Holt P.S., Davies R.H., Dewulf J., Gast R.K., Huwe J.K., Jones D.R., Waltman D., Willian K.R. (2011). The Impact of Different Housing Systems on Egg Safety and Quality. Poult. Sci..

[13] Sauvant D., Schmidely P., Daudin J.J., St-Pierre N.R. (2008). Meta-Analyses of Experimental Data in Animal Nutrition. Animal.

[14] Page M.J., McKenzie J.E., Bossuyt P.M., Boutron I., Hoffmann T.C., Mulrow C.D., Shamseer L., Tetzlaff J.M., Akl E.A., Brennan S.E. (2021). The PRISMA 2020 Statement: An Updated Guideline for Reporting Systematic Reviews. BMJ.

[15] Wallace B.C., Lajeunesse M.J., Dietz G., Dahabreh I.J., Trikalinos T.A., Schmid C.H., Gurevitch J. (2017). OpenMEE: Intuitive, Open-Source Software for Meta-Analysis in Ecology and Evolutionary Biology. Methods Ecol. Evol..

[16] DerSimonian R., Laird N. (2015). Meta-Analysis in Clinical Trials Revisited. Contemp. Clin. Trials.

[17] Koricheva J., Gurevitch J., Mengersen K. (2013). Handbook of Meta-Analysis in Ecology and Evolution.

[18] Palupi E., Jayanegara A., Ploeger A., Kahl J. (2012). Comparison of Nutritional Quality between Conventional and Organic Dairy Products: A Meta-Analysis. J. Sci. Food Agric..

[19] Higgins J.P.T., Thompson S.G., Deeks J.J., Altman D.G. (2003). Measuring Inconsistency in Meta-Analyses Testing for Heterogeneity. BMJ.

[20] Borenstein M., Hedges L.V., Higgins J.P.T., Rothstein H.R. (2021). Introduction to Meta-Analysis.

[21] Albarki H.R., Kusuma R.I., Daulai M.S., Suntara C., Iwai C.B., Jayanegara A., Cherdthong A. (2024). Effects of Rumen-Protected Fat on Rumen Fermentation Products, Meat Characteristics, Cattle Performance, and Milk Quality: A Meta-Analysis. Anim. Feed Sci. Technol..

[22] Ogbuewu I.P., Okoro V.M., Mbajiorgu C.A. (2020). Probiotic-Yeast Improves Performance Indicators in Broiler Chickens: Evidence from Meta-Analysis. Appl. Ecol. Environ. Res..

[23] Yano A.A., Astuti D., Respati A.N., Ningsih N., Triswanto, Purnamayanti L., Gao M., Rahman M.A., Abdel-Moneim A.M.E., Elsadek M.F. (2025). A Meta-Analysis to Study the Effects and Relationships of Various Selenium Sources and Forms on Production Performance, Antioxidant Status and Egg Quality of Laying Hens. J. Sci. Food Agric..

[24] Rosenberg M.S. (2005). The File-Drawer Problem Revisited: A General Weighted Method for Calculating Fail-Safe Numbers in Meta-Analysis. Evolution.

[25] Gakhar N., Goldberg E., Jing M., Gibson R., House J.D. (2012). Effect of Feeding Hemp Seed and Hemp Seed Oil on Laying Hen Performance and Egg Yolk Fatty Acid Content: Evidence of Their Safety and Efficacy for Laying Hen Diets. Poult. Sci..

[26] Konca Y., Yuksel T., Yalcin H., Beyzi S.B., Kaliber M. (2019). Effects of Heat-Treated Hempseed Supplementation on Performance, Egg Quality, Sensory Evaluation and Antioxidant Activity of Laying Hens. Br. Poult. Sci..

[27] Mierliţă D. (2019). Fatty Acids Profile and Oxidative Stability of Eggs from Laying Hens Fed Diets Containing Hemp Seed or Hempseed Cake. S. Afr. J. Anim. Sci..

[28] Mierlita D., Teușdea A.C., Matei M., Pascal C., Simeanu D., Pop I.M. (2024). Effect of Dietary Incorporation of Hemp Seeds Alone or with Dried Fruit Pomace on Laying Hens’ Performance and on Lipid Composition and Oxidation Status of Egg Yolks. Animals.

[29] Neijat M., Gakhar N., Neufeld J., House J.D. (2014). Performance, Egg Quality, and Blood Plasma Chemistry of Laying Hens Fed Hempseed and Hempseed Oil. Poult. Sci..

[30] Neijat M., Suh M., Neufeld J., House J.D. (2016). Increasing Levels of Dietary Hempseed Products Leads to Differential Responses in the Fatty Acid Profiles of Egg Yolk, Liver and Plasma of Laying Hens. Lipids.

[31] Neijat M., Suh M., Neufeld J., House J.D. (2016). Hempseed Products Fed to Hens Effectively Increased N-3 Polyunsaturated Fatty Acids in Total Lipids, Triacylglycerol and Phospholipid of Egg Yolk. Lipids.

[32] Park S.-O., Hwangbo J., In-Suh Y., Park B.-S. (2014). Gamma-Linolenic Acid Egg Production Enriched with Hemp Seed Oil and Evening Primrose Oil in Diet of Laying Hens. J. Environ. Biol..

[33] Raza T., Chand N., Khan R.U., Shahid M.S., Abudabos A.M. (2016). Improving the Fatty Acid Profile in Egg Yolk through the Use of Hempseed (*Cannabis sativa*), Ginger (*Zingiber officinale*), and Turmeric (*Curcuma longa*) in the Diet of Hy-Line White Leghorns. Arch. Anim. Breed..

[34] Shahid S., Chand N., Khan R.U., Suhail S.M., Khan N.A. (2015). Alternations in Cholesterol and Fatty Acids Composition in Egg Yolk of Rhode Island Red x Fyoumi Hens Fed with Hemp Seeds (*Cannabis sativa* L.). J. Chem..

[35] Silversides F.G., Lefrançois M.R. (2005). The Effect of Feeding Hemp Seed Meal to Laying Hens. Br. Poult. Sci..

[36] Skřivan M., Englmaierová M., Vít T., Skřivanová E. (2019). Hempseed Increases Gamma-Tocopherol in Egg Yolks and the Breaking Strength of Tibias in Laying Hens. PLoS ONE.

[37] Taaifi Y., Belhaj K., Mansouri F., Rbah Y., Melhaoui R., Houmy N., Ben Moumen A., Azeroual E., Addi M., Elamrani A. (2023). The Effect of Feeding Laying Hens with Nonindustrial Hemp Seed on the Fatty Acid Profile, Cholesterol Level, and Tocopherol Composition of Egg Yolk. Int. J. Food Sci..

[38] Halle I., Schöne F. (2013). Influence of Rapeseed Cake, Linseed Cake and Hemp Seed Cake on Laying Performance of Hens and Fatty Acid Composition of Egg Yolk. J. Fur Verbraucherschutz Und Lebensmittelsicherheit..

[39] Mierlita D., Daraban S., Teușdea A.C., Stanciu A.S. (2024). Effect of Dietary Cold-Pressed Hempseed Cake Supplemented with Tomato Waste on Laying Hen Performance and Egg Yolk Lipid Profile and Antioxidant Status Before and After Storage. Animals.

[40] Lanzoni D., Skřivan M., Englmaierová M., Petrosillo E., Marchetti L., Skřivanová V., Bontempo V., Rebucci R., Baldi A., Giromini C. (2025). Effects of Dietary Hemp Co-Product Inclusion on Laying Hens Performances and on Egg Nutritional and Functional Profile. Ital. J. Anim. Sci..

[41] Cohen J. (1988). Statistical Power Analysis for the Behavioral Sciences.

[42] Swanson D., Block R., Mousa S.A. (2012). Omega-3 Fatty Acids EPA and DHA: Health Benefits throughout Life. Adv. Nutr..

[43] Mozaffarian D., Wu J.H.Y. (2011). Omega-3 Fatty Acids and Cardiovascular Disease: Effects on Risk Factors, Molecular Pathways, and Clinical Events. J. Am. Coll. Cardiol..

[44] Erhardt R., Cardoso B.R., Meyer B.J., Brownell S., O’Connell S., Mirzaee S., Duckham R.L., Macpherson H. (2021). Omega-3 Long-Chain Polyunsaturated Fatty Acids: Are They Beneficial for Physical and Cognitive Functioning in Older Adults?. J. Nutr. Health Aging.

[45] Jacques C., Levy E., Muckle G., Jacobson S.W., Bastien C., Dewailly É., Ayotte P., Jacobson J.L., Saint-Amour D. (2011). Long-Term Effects of Prenatal Omega-3 Fatty Acid Intake on Visual Function in School-Age Children. J. Pediatr..

[46] Lorente-Cebrián S., Costa A.G.V., Navas-Carretero S., Zabala M., Laiglesia L.M., Martínez J.A., Moreno-Aliaga M.J. (2015). An Update on the Role of Omega-3 Fatty Acids on Inflammatory and Degenerative Diseases. J. Physiol. Biochem..

[47] Saglimbene V.M., Wong G., van Zwieten A., Palmer S.C., Ruospo M., Natale P., Campbell K., Teixeira-Pinto A., Craig J.C., Strippoli G.F.M. (2020). Effects of Omega-3 Polyunsaturated Fatty Acid Intake in Patients with Chronic Kidney Disease: Systematic Review and Meta-Analysis of Randomized Controlled Trials. Clin. Nutr..

